# Assessment of the Long-Term Mental Health Effects on Austrian Students after COVID-19 Restrictions

**DOI:** 10.3390/ijerph192013110

**Published:** 2022-10-12

**Authors:** Stefan Kaltschik, Christoph Pieh, Rachel Dale, Thomas Probst, Barbara Pammer, Elke Humer

**Affiliations:** 1 Department for Psychosomatic Medicine and Psychotherapy, University for Continuing Education Krems, 3500 Krems, Austria; 2 Independent Researcher, 8010 Graz, Austria

**Keywords:** adolescents, COVID-19, depression, anxiety, insomnia, stress, health behaviors

## Abstract

The mental health of adolescents has been severely affected by the COVID-19 pandemic. The aim of this study was to assess the mental health of Austrian adolescents in spring 2022, a time during which COVID-19-related restrictions had been significantly lifted. A total of N = 616 students aged between 14 and 20 participated in a cross-sectional survey between April and May 2022 (t2). The prevalence of clinically relevant symptoms was 73% among girls and 44% among boys for depression (PHQ-9 score ≥ 11), 57% in girls and 35% in boys for anxiety (GAD-7 score ≥ 11), 34% in girls and 21% in boys for sleeping problems (ISI score ≥ 15), and 95% in girls and 81% in boys for experiencing at least moderate stress (PSS-10 score ≥ 14). Frequent suicidal ideations were reported by 24% of girls and 12% of boys. These results were compared with the results from a cross-sectional study from February 2021 (t1). To account for differences in covariates between samples, data were propensity score matched before the analysis. Compared with t1, we found an increase among girls regarding clinically relevant symptoms of depression (OR = 1.78), anxiety (OR = 1.34), insomnia (OR = 1.63), and suicidal ideations (OR = 1.96; *p* < 0.05 for all measures). Significant correlations were found between smartphone use and mental health and physical activity and mental health for both genders. The results of this study indicated that even during the third year of the COVID-19 pandemic, the mental health of adolescents in Austria is still severely impaired.

## 1. Introduction

With the outbreak of the coronavirus disease 2019 (COVID-19) pandemic, Austrian school students experienced several episodes of school closures as a part of general containment measures to slow the spread of the virus. Consequently, students had to cope with the challenges of distance learning. These measures resulted in reductions in important mental health protective factors (e.g., social contacts, daily structure) and increased stress [1,2]. 

Studies showed that mental health in the general Austrian population has been declining continuously since the beginning of the pandemic [3,4,5]. Adolescents in particular showed severe deterioration in mental health. While meta-analyses showed that prevalence estimates differed considerably between studies, significant mental health declines seem to be robust [6,7].

The impact of remote schooling on mental health has been the subject of various recent studies. While positive effects on family cohesion, personal growth, and sleep duration were discussed, these were mostly diminished by reduced coping opportunities, leading to a decline in well-being and mental health, increased feelings of loneliness, increased potential for family conflicts, and decreased school performance [8,9,10,11,12,13]. A qualitative study of Austrian students using content analysis reported school-related concerns and concerns about restrictions as the most burdensome, while social and recreational activities were mentioned as the strongest protective sources [14].

An increase in mental health problems in adolescents during the COVID-19 pandemic was reported by several systematic reviews [7,15]. A meta-analysis that examined the global prevalence of depression and anxiety symptoms in children and adolescents showed a prevalence of 25% for depression and 20% for anxiety symptoms, while the prevalence was higher in girls and in more recent studies [7]. Overall, the high variability in the prevalences between the included studies must be considered. Differences in sample sizes, mental health definitions, instruments to evaluate mental health, differing age ranges, and regional aspects seemed to influence the reported prevalences [15]. 

For Austria, a first study conducted at the end of the first year of the COVID-19 pandemic (February 2021) during an extended episode of remote schooling showed a higher prevalence of depressive symptoms, anxiety symptoms, sleep-related problems, and suicidal thoughts than before the pandemic [16]. A first follow-up study from 19 June to 2 July 2021, after one semester of distance learning, reported a slight improvement in mental health, albeit still at a lower level than before the pandemic [17]. This promising trend could not be replicated in a study from 14 September to 14 November 2021 [18]. Girls’ mental health had declined, while boys’ mental health remained at the same low levels as in February 2021. The stronger effect on girls appeared to be robust in these studies and is well documented in the literature [19].

Several studies indicated that smartphone use and physical activity appear to be associated with mental health [20,21,22,23]. While excessive smartphone use tends to be associated with negative consequences [20,21], physical activity is often described as a protective factor [22,23]. Smartphone use is particularly common among young people. Consequently, when studying adolescent mental health, both the frequency of smartphone use and the frequency of physical activity should be monitored.

Students have now returned to regular classes since the beginning of the school year 2021/2022. Gradually most general restrictions have been lifted and since April 2022, with minor exceptions, social and leisure activities that serve as protective factors for mental health can be experienced again.

The main objective of this work was to assess the mental health of Austrian students in spring 2022, the third year of the COVID-19 pandemic. Specifically, we aimed to investigate the prevalence of depressive symptoms, anxiety symptoms, sleep problems, and perceived stress. As gender differences in mental health are well documented, we also attempted to focus on gender effects. A secondary objective of this study was to test the hypothesis that mental health has improved since the beginning of the COVID-19 pandemic. Therefore, we compared the results of this study with the results of a previous study from the first year of the pandemic [16]. This study also explored the associations of smartphone usage and physical activity with adolescents’ mental health. This research is a further step toward a deeper understanding of the long-term effects of the COVID-19 pandemic and its measures on adolescents’ mental health in Austria. 

## 2. Materials and Methods

We used the STROBE guidelines [24] to report this cross-sectional study.

### 2.1. Study Design

We conducted a cross-sectional online study to determine the mental health statuses of Austrian students between the ages of 14 and 20. Study data were collected and managed using REDCap (Research Electronic Data Capture) hosted at servers of the University of Continuing Education Krems [25,26]. Data were collected between 26 April and 24 May 2022. At this time, the general COVID-19 measures in Austria were eased and students attended classes regularly. To examine the development of mental-health-related symptoms between the first year of the pandemic and the third year of the pandemic, the data from this study (t2) were compared with a matched sample from a previous survey conducted between 3 February 2021 and 28 February 2021 (t1). A detailed analysis of this earlier study (t1) was provided elsewhere [16]. Data that were part of the already published work [16] were only used for comparison reasons in the current study.

### 2.2. Participants

Participants were recruited through online convenience sampling. School representatives were contacted and asked to forward an invitation with the link to the online survey to students. Participants were eligible if (a) their age was between 14 and 20 years, (b) they attended an Austrian school, and (c) they provided electronic informed consent. Participants then completed a survey consisting of demographic data and mental health questionnaires. Only individuals who provided information on all outcome variables were included in the statistical analysis.

### 2.3. Variables

#### 2.3.1. Well-Being

The German version of the WHO-5 Index [27,28] was used to assess the general well-being of respondents. The WHO-5 index consists of 5 self-rating items on a 6-point Likert scale. Each item is scaled from 0 (never) to 5 (most of the time). A summed score of all items results in a score ranging from 0 (absence of well-being) to 25 (highest well-being) is calculated. The scores were multiplied by four to translate them into a percentage scale from 0 to 100. At t2, Cronbach’s alpha was 0.83 for girls and 0.80 for boys.

#### 2.3.2. Stress

The German version of the Perceived Stress Scale (PSS-10) [29] was used to assess the perceived stress levels of the participants. The PSS-10 is a valid, reliable, and economical instrument for assessing perceived stress [29]. It consists of 10 self-rating items on a 5-point Likert scale. A general score is calculated as a sum score of the individual items ranging from 0 (lowest possible perceived stress level) to 40 (highest perceived stress level). We used a cut-off score of 14 to define moderate stress. At t2, Cronbach’s alpha was 0.84 for girls and 0.87 for boys.

#### 2.3.3. Depression

Depressive symptoms were assessed with the Patient Health Questionnaire-9 (PHQ-9) [30]. The PHQ-9 consists of nine items representing the DSM5 criteria for depression. Each item is scaled from 0 (not at all) to 3 (nearly every day) resulting in a sum score from 0 to 27. The cut-off for clinically relevant depressive symptoms in adolescents is 11 points [31]. At t2, Cronbach’s alpha was 0.86 for girls and 0.88 for boys.

#### 2.3.4. Suicidal Ideation

Suicidal thoughts were assessed with item nine of the PHQ-9. This 4-point Likert scaled item asks: “Over the last two weeks, how often have you been bothered by thoughts that you would be better off dead or of hurting yourself in some way?” Answers are coded as 0 (not at all), 1 (several days), 2 (more than half the days), and 3 (nearly every day). We set a cutoff for severe suicidal ideations at ≥2.

#### 2.3.5. Anxiety

General anxiety was measured with the German version of the Generalized Anxiety Disorder-7 (GAD-7) [32,33]. The GAD-7 is a 7-item, four-point Likert-scaled questionnaire. Items range from 0 (not at all) to 3 (nearly every day). The sum score lies between 0 and 21 with a cutoff for clinically relevant general anxiety symptoms of 11 points [32]. At t2, Cronbach’s alpha was 0.86 for girls and 0.89 for boys.

#### 2.3.6. Insomnia

Sleep-related problems were measured with the Insomnia Severity Index (ISI) [34]. The ISI consists of seven four-point Likert-scale items with a maximum score of 28. The cut-off for moderate insomnia is 15 points [35]. At t2, Cronbach’s alpha was 0.828 for girls and 0.831 for boys.

#### 2.3.7. Smartphone Usage

The frequency of smartphone usage was assessed on a 6-point scale ranging from 1 to 6 as follows: 1: <1 h/day, 2: 1–2 h/day, 3: 3–4 h/day, 4: 5–6 h/day, 5: 7–8 h/day, and 6: >8 h/day. Mobile phone use was queried identically as in previous studies (i.e., the Health Behaviour in School-Aged Children Study) conducted in Austria prior to this survey [16,36] in order to allow for comparison.

#### 2.3.8. Physical Activity

Physical activity was assessed on a scale from 0–7, indicating the number of days per week with more than 60 min of physical activity. We set the reference of 60 min per day, as this reference was also used in previous studies conducted on Austrian adolescents [16,36] and is used in WHO recommendations on physical activity for health [37] in order to allow for comparison.

#### 2.3.9. Gender

Participants were asked to indicate their gender category as boy, girl, or diverse. Diverse indicates students whose gender identity or gender expression does not conform to socially defined male or female gender norms.

### 2.4. Study Sample Size

We calculated the required sample size for performing a linear regression analysis for within measures (matched pairs: t1 vs. t2), between measures (gender), and the interaction term with G-Power [38]. To detect a medium standardized effect (d = 0.5) with 80% power and 5% α error probability, a total sample size of 128 participants was needed. For the *chi*^2^ tests with 1 degree of freedom (2 × 2 table), 80% power, and 5% α error probability to detect a medium standardized effect, G-Power indicated a required sample size of 88 participants.

### 2.5. Statistical Analyses

To answer our primary research question, mean values and standard deviations were calculated for all continuous outcomes. Frequencies indicating the prevalence of specific symptoms were calculated for dichotomous outcomes (0—below the cutoff, 1—over the cutoff). As differences between gender were to be expected, we reported all results in a disaggregated form. To study the gender differences in the outcomes, we used a linear regression model with gender as a dummy coded independent variable (girls—0, boys—1) and the scores of the mental health questionnaires as dependent variables.

To assess differences in the mental health scores between t2 and t1, we added a dummy coded time variable (t1—0, t2—1) as an additional independent variable to the regression model. We also included the interaction term of gender and time in the regression model to test the hypothesis of whether changes in scores differed over time with respect to gender.

To test whether the frequencies of the dichotomous outcomes differed from t1, we calculated McNemar’s chi^2^ tests [39] and McNemar’s odds ratios.

Because we were not able to match data at the individual level due to data protection issues, it was not possible to perform a longitudinal analysis to answer our secondary hypothesis. Thus, we used a repeated cross-sectional design to compare the results from t2 to t1. Since we found significant differences in the covariates between the two samples, we used propensity score matching to reach a covariate balance between the samples. The covariates considered for the matching were gender, age, region, and migration background. Propensity score matching was performed with the MatchIt package in R [40]. After assessing different matching methods, nearest neighbor matching without replacement yielded the best reduction in the standardized differences in the covariates. The matching performance between different methods was evaluated by comparing the reduction in standardized mean differences and variance ratios using visualizations. All standardized mean differences between the covariates of the two samples were less than 0.01 after matching, indicating a good covariate balance (Supplementary Figure S1). Propensity score matching resulted in matched pair data; therefore, we used statistical methods for repeated measures for all comparisons over time.

To study the relationship between smartphone usage and mental health scores and physical activity and mental health scores, we calculated Spearman’s rank correlation coefficient (*rho*). To analyze whether the correlation coefficients differed between t1 and t2, we used Fisher’s z-transformation before using a z-test. [41].

To test whether there was a significant difference in smartphone usage and physical activity between t1 and t2, we used the Wilcoxon signed-rank test.

Significance levels were set at the 5% level. Estimations of effects for continuous variables after matching were performed with R, and all other analyses were computed using SPSS 27 (SPSS Inc., Chicago, IL, USA).

Cases with missing data were excluded listwise from the analysis.

## 3. Results

### 3.1. Sample

**Figure 1:**
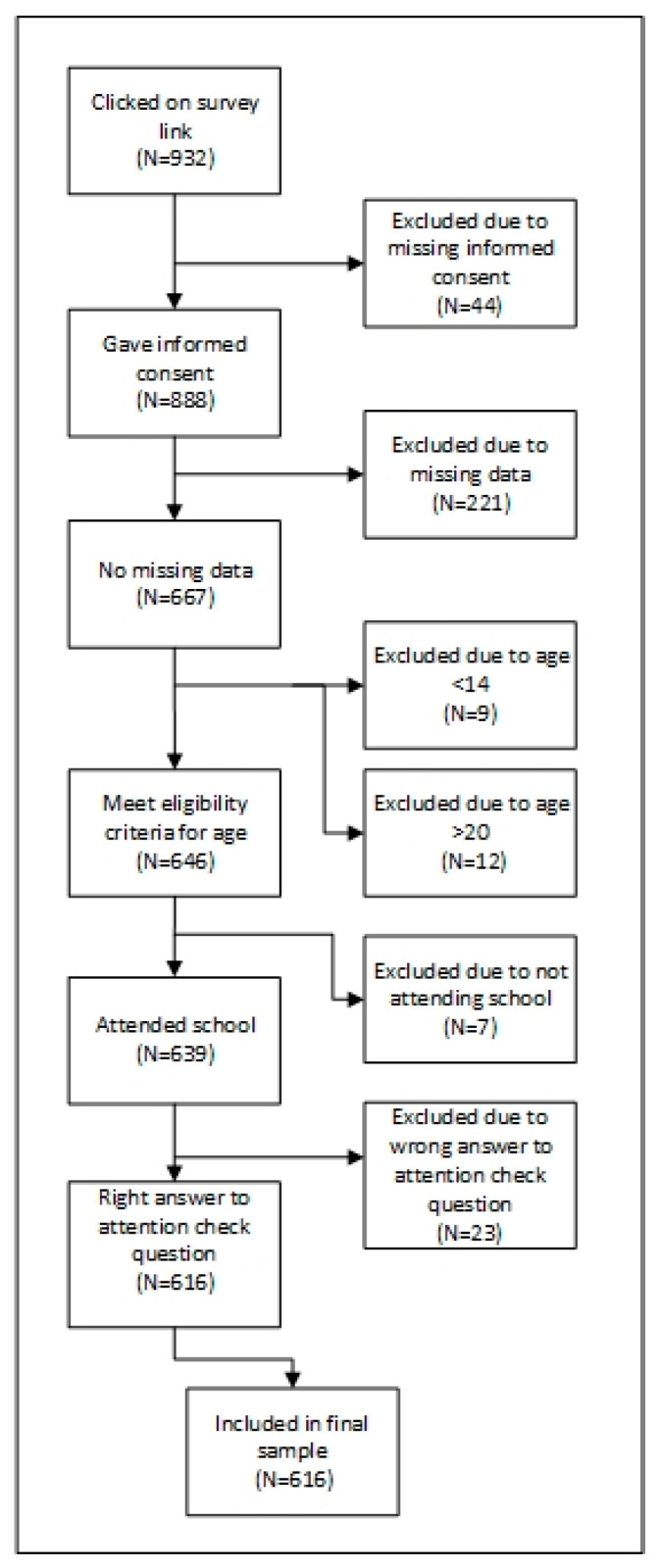
Flow diagram of the process from survey participation to study inclusion.

Following the American Association for Public Opinion Research (AAPOR) reporting guidelines for non-probability online samples [42], we report that 71.6% of those who clicked on the survey link completed the entire survey, resulting in a 71.6% participation rate (completion rate). Figure 1 describes the flow of participants. A total of 616 subjects completed the survey that satisfied the inclusion criteria, of whom 477 were girls and 17 identified their gender category as diverse. Since the small sample size of participants describing themselves as diverse turned out to be suboptimal for statistical analysis and would have affected the matching procedure, they were not considered in the further analysis. The descriptive data (unmatched) for the diverse gender category can be found in Supplementary Table S1.

The matching process resulted in a total sample size of 1198 participants (599 for each sample). The sample for t1 consisted of 481 girls and 118 boys and for t2 of 477 girls and 122 boys. Descriptive statistics for both matched samples are shown in Table 1.

### 3.2. Mental Health Measures

The results for all outcomes are presented in Table 2. When considering the clinically relevant results exceeding the specified cutoffs for the respective measures, we found a prevalence of clinically relevant symptoms (exceeding the cutoff) of depression of 73% in girls and 44% in boys. Anxiety symptoms were clinically relevant in 57% of girls and 35% of boys. In total, 34% of the girls and 21% of the boys reported sleeping problems. Suicidal thoughts on more than half of the days were reported by 24% of girls and 12% of boys. In total, 95% of girls and 81% of boys showed at least a moderate level of perceived stress.

All continuous mental health measures (scores of the WHO5, PHQ-9, GAD-7, ISI, and PSS-10) were affected by gender and time (all *p* < 0.001, see Supplementary Table S2), whereas no significant interaction effects between gender and time were observed. Girls showed lower mental health scores than boys and their mental health was lower at t2 (see Table 2).

**Figure 2:**
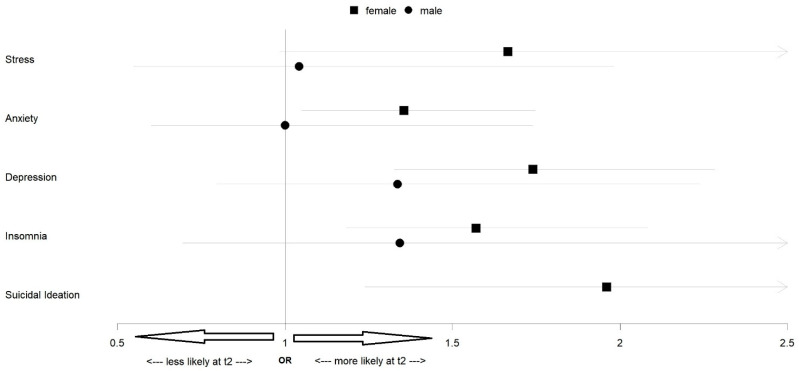
McNemar’s odds ratios (ORs) for clinically relevant symptoms of stress, anxiety, depression, insomnia, and suicidal ideation at t2 versus t1. Stress: PSS-10 score ≥ 14, anxiety: GAD-7 score ≥ 11, depression: PHQ-9 score ≥ 11, insomnia: ISI score ≥ 15, suicidal ideation: PHQ-9 item 9 score ≥ 2. The *x*-axis represents the odds ratios (ORs): <1—clinically relevant symptoms less likely at t2; >1—clinically symptoms more likely at t2. A 95% confidence interval (CI, indicated as grey lines) overlapping 1 indicates a non-significant difference between t1 and t2. The OR for suicidal ideations among boys could not be calculated due to zero events in necessary cells.

Figure 2 depicts the differences in the odds of reporting clinically relevant symptoms between t1 and t2, separately for girls and boys. Odds for scoring over the cutoff for depression (OR = 1.78, 95% CI [1.34, 2.44]), anxiety (OR = 1.34, 95% CI [1.02, 1.80]), insomnia (OR = 1.63, 95% CI [1.21, 2.25]), and suicidal ideation [OR = 1.96, 95% CI [1.24, 3.39]) were higher at t2 vs. t1 in the girl’s sample. No significant differences in these measures between the two surveys were found in the boy’s sample. Detailed results are provided in Supplementary Table S3.

### 3.3. Smartphone Usage

Descriptive results for smartphone use by gender and time point are presented in Table 3. Among girls, smartphone use was lower at t2 (mdn = 3) than at t1 (mdn = 4), *z* = −3.975, *p* < 0.001. Smartphone use also decreased from t1 (mdn = 4) to t2 (mdn = 3) among boys, *z* = −2.527, *p* < 0.001. The correlation analysis (Table 4) showed significant correlations between smartphone usage and mental health at t2.

For girls, smartphone use was negatively associated with well-being (*rho*(475) = −0.213, *p* < 0.001) and increased smartphone usage was associated with an increase in depression symptoms (*rho*(475) = 0.214, *p* < 0.001), general anxiety (*rho*(475) = 0.135, *p* < 0.001), insomnia (*rho*(475) = 0.185, *p* < 0.001), and perceived stress (*rho*(475) = 0.144, *p* < 0.001). For boys, the correlation between smartphone use and well-being was *rho*(120) = −0.235, *p* < 0.05, and smartphone use was associated with increased symptoms regarding depression (*rho*(120) = 0.335, *p* < 0.001), insomnia (*rho*(120) = 0.353, *p* < 0.001), and perceived stress (*rho*(120) = 0.311, *p* < 0.001). There was no significant correlation with general anxiety symptoms in boys.

At t1, smartphone usage correlated with mental health variables in girls (Supplementary Table S4) as well as boys (Supplementary Table S5).

Compared with t1, the association between smartphone use and insomnia decreased at t2 among girls (*z* = 2.143, *p* = 0.016). For boys, the relationship with anxiety symptoms declined significantly (*z* = 2.2152, *p* = 0.016). All other comparisons revealed no significant differences between the studies. A detailed list with all comparisons can be found in Supplementary Tables S6 and S7.

### 3.4. Physical Activity

Table 4 summarizes the descriptive data on the number of days with at least 60 min of physical activity in boys and girls at t1 and t2. Girls showed a median physical activity of 2 days per week. Compared with t1 (mdn = 3 days/week), this was significantly lower (*z* = −3.472, *p* = 0.001). Boys showed a median physical activity of 3 days per week. There was no significant difference compared with t1.

Correlation analysis showed significant correlations between physical activity and mental health (Table 5). For girls’ physical activity at t2 was positively associated with well-being, *rho* = 0.264, *p* < 0.01. All other mental health measures showed significant negative correlations with physical activity (Table 4). At t2, boys also showed a positive correlation between physical activity and well-being (*rho* = 0.235, *p* = 0.01) and negative correlations for general anxiety and stress (Table 5).

At t1, physical activity correlated with all mental health variables in girls (Supplementary Table S4). With the exception of the insomnia severity index, all mental health indicators were associated with physical activity in boys at t1 (Supplementary Table S5). Compared with t1, for girls, the correlation between physical activity and the ISI score declined (*z* = 2.14. *p* = 0.016). There were no differences in the other correlations. For boys, only the correlation between depression and physical activity decreased significantly between t1 and t2 (*z* = −2.35, *p* = 0.09).

## 4. Discussion

This study showed that despite a major easing of coronavirus-related restrictions in the third year of the pandemic, the mental health of adolescents was still severely impaired. The results showed a significant decline in most domains of mental health among adolescents, while girls’ mental health was significantly worse than boys’. This supported the hypothesis of negative long-term effects being caused by the COVID-19 pandemic and showed that the lifting of restrictions has failed to return mental health to pre-pandemic levels.

When comparing the results of our study to the findings of t1, we found significant decreases in mental health for all continuous outcomes. As no significant interaction effects between gender and time were found, we concluded that girls and boys seemed to be equally affected by the decline in mental health between t1 and t2. This also supported the hypothesis of negative long-term effects being caused by the COVID-19 pandemic and showed that the lifting of restrictions has failed to return mental health to pre-pandemic levels.

Almost two-thirds of girls in our study showed signs of clinically relevant depression and 24% had suicidal ideations on more than half of the days. The odds for suicidal ideations more than doubled for girls compared with t1, which is an alarming development. However, the prevalence in boys is also a concern.

In general, these results agreed well with existing studies on gender differences in adolescent mental health during the pandemic [19,43,44]. To explain these gender effects, one hypothesis claims that girls appear to be more affected by the COVID-19 pandemic due to their stronger reliance on a social network for emotional support. The restrictions imposed by COVID-19 severely reduced social contact, leading to the loss of a key source of supply [44]. Following this logic, however, our results contradict this hypothesis, as most social distancing restrictions were lifted by spring 2022, suggesting that mental health should have recovered since then. Another explanation might consider gender differences in physical activity as a relevant factor. Moreover, there is strong evidence of a link between physical activity and mental health [45], and studies showed that boys are more physically active than girls [46]. Boys, in particular, are also more likely to participate in organized sports, which could explain the gender differences in mental health through the frequency of physical activity [47]. Furthermore, sports clubs add a social domain to physical activity, which can serve as an additional protective factor that is available mainly to boys. In our study, we found that boys physical activity stayed at the same level as at t1, while girls’ physical activity was lower.

The pandemic increased smartphone usage [48]. Increased smartphone usage is associated with decreased academic performance, physical health problems, sleep problems, and mental health problems [20]. Consistent with the literature, we found higher smartphone use among girls. Smartphones allow for social connectedness and may serve as a coping strategy in episodes of increased stress. In particular, the use of social media as a coping strategy tends to harm the psyche and is more likely to occur in girls [49]. Since we did not ask about the actual content of smartphone usage, we can only speculate whether this also applies to our study. Compared with t1, smartphone use was significantly lower at t2. This may be explained by the lifting of most COVID-19-related restrictions, allowing adolescents to spend more time with other activities that were not available during the episodes with stronger restrictions. Nevertheless, our study showed that the association between smartphone use and mental health, and between physical activity and mental health, remained on the same level as at t1. In both studies, increased smartphone use was associated with lower mental health, while physical activity was associated with better mental health. We observed these correlations across most mental health domains in both genders. Although the correlations were in the small to moderate range, they were robust in magnitude and direction.

While boys may use physical activity as a coping mechanism, girls may rely more on smartphone usage to cope with stressful situations. As physical activity is associated with positive outcomes and smartphone usage with negative outcomes, this may also be one explanation for the gender differences. Although beyond the scope of this study, the causal mechanisms between gender and smartphone use, physical activity, and mental health appear to represent a worthwhile direction for future work.

Unfortunately, it is not possible to isolate the impact of the COVID-19 pandemic from other influential events that affected youth at the time of the study. Shortly before the start of the survey, the Russian–Ukrainian conflict escalated, and young people were confronted with disturbing news from a neighboring European country. Additionally, the climate crisis may play a part in generating a situation of an uncertain and unstable future, which can lead to climate anxiety [50]. Adolescents who are heavy users of social media, especially girls, may be particularly affected.

Some methodological limitations of our study need to be mentioned. A major drawback was the overrepresentation of female adolescents. The low number of boys may have contributed to the insignificant changes in mental health between time points in boys. Furthermore, data could not be matched at the individual level between time points due to the strictly anonymous data collection. The propensity score matching process does not replace random sampling and since only a limited set of confounders can be integrated into the analysis, the risk of bias from unobserved confounders should be taken into consideration. The cross-sectional design of this study did not allow us to draw causal inferences and generalizations to broader populations must be made with caution. A bias caused by self-selection by the participants must also be taken into account. Another drawback is that the mental health estimates were based on self-rating instruments and not validated by structured clinical interviews.

## 5. Conclusions

The lifting of the COVID-19 restrictions did not lead to an improvement in the mental health of adolescents in Austria. Mental health levels were still at a low level and had been declining since the early stages of the pandemic. As expected, the results revealed gender differences in terms of mental health, as well as associations of mental health with health behaviors. We concluded that there needs to be a broad discussion about gender-specific interventions and measures to counteract this trend in order to halt further declines in adolescent mental health. Future research should also consider the impact of stressors that are not directly related to the COVID-19 pandemic (e.g., Ukraine crisis, climate crisis). As we found that physical activity and smartphone usage were associated with mental health, these factors should be considered when developing interventions.

This study provided an update on the mental health status of Austrian adolescents and is an important contribution to the understanding of the long-term effects of the COVID-19 pandemic and associated containment efforts on adolescent mental health.

## Figures and Tables

**Table 1:** Matched study sample characteristics.

	t1 ^a^		t2 ^b^		Total	
	n	%	n	%	n	%
Gender						
Girl	481	80.3	477	79.6	958	80
Boy	118	19.7	122	20.4	240	20
Migration background						
No	491	82.0	488	81.5	979	81.7
Yes	108	18.0	111	18.5	219	18.3
Region						
Northeast	380	63.4	379	63.3	759	63.4
Southeast	171	28.5	172	28.7	343	28.6
West	48	8	48	8	96	8
Age M (SD)	16.64 (1.46)		16.66 (1.48)		16.65 (1.47)	

^a^ t1: sample from February 2021; ^b^ t2: sample from April to May 2022.

**Table 2:** Statistics for the mental health questionnaires for the matched samples by gender and time.

Total	Girls		Boys		
	t1	t2	t1	t2	Statistics
**N**	481	477	118	122	
**WHO-5** M (SD)	36.62 (18.95)	32.69 (18.71)	44.61 (23.07)	44.03 (20.60)	F(2;1195) = 27.78; *p* < 0.001
**PHQ-9** M (SD)	11.99 (6.02)	14.16 (6.18)	9.19 (6.55)	10.70 (6.53)	F(2;1195) = 27.78; *p* < 0.001
≥11, % (n)	60 (290)	73 (346)	37 (44)	44 (54)	
**GAD-7** M (SD)	10.80 (4.96)	11.48 (5.04)	8.05 (5.22)	8.56 (5.52)	F(2;1195) = 32.32; *p* < 0.001
≥11, % (n)	50 (238)	57 (272)	35 (41)	35 (43)	
**ISI** M (SD)	10.85 (5.31)	11.89 (6.20)	8.26 (5.67)	9.55 (6.16)	F(2;1195) = 22.39; *p* < 0.001
≥15, %(n)	24 (117)	34 (160)	16 (19)	21 (25)	
**PSS-10** M (SD)	24.35 (6.83)	24.90 (6.61)	20.20 (7.90)	20.02 (7.70)	F(2;1195) = 40.90; *p* < 0.001
≥14, % (n)	92 (442)	95 (453)	81 (95)	81 (99)	
**Suicidal ideations**					
≥“more than half the days” % (n)	12 (59)	24 (114)	10 (12)	12 (15)	

t1: Sample from February 2021 (after one semester of remote schooling); sample from April to May 2022 (school opened and most restrictions lifted). M: mean score, SD: standard deviation, F: test statistics of the GLM models; WHO-5: World Health Organization Wellbeing Index, PHQ-9: Patient Health Questionnaire 2 Scale, GAD-7: General Anxiety Disorder 7 Scale, ISI: Insomnia Severity Index Score, PSS-10: Perceived Stress Scale 10. Suicidal ideations: item 9 of the PHQ-9: “Thoughts that you would be better off dead, or of hurting yourself”.

**Table 3:** Smartphone use by gender and time (t1 and t2).

		Smartphone Use					
		<1 h/Day	1–2 h/Day	3–4 h/Day	5–6 h/Day	7–8 h/Day	>8 h/Day
Boys	t1	0.8% (1)	11.9% (14)	31.4% (37)	30.5% (36)	15.3% (18)	10.2% (12)
	t2	0.8% (1)	19.7% (24)	47.5% (58)	16.4% (20)	7.4% (9)	8.2% (10)
Girls	t1	1% (5)	10.2% (49)	32.8% (158)	25.2% (121)	16.6% (80)	14.1% (68)
	t2	1.5% (7)	13.4% (64)	38.8% (185)	26.4% (126)	11.5 (55)	8.4% (40)

**Table 4:** Physical activity by gender and time (t1 and t2).

		Physical Activity							
	Days/Week	0	1	2	3	4	5	6	7
Boys	t1	11.9% (14)	12.7% (15)	16.1% (19)	10.2% (12)	11.9% (14)	11.9% (14)	5.9% (7)	19.5% (23)
	t2	11.5% (14)	13.9% (17)	19.7% (24)	18.9% (23)	9% (11)	8.2% (10)	9.8% (12)	9% (11)
Girls	t1	7.1% (34)	14.3% (69)	19.1% (92)	20.2% (97)	16% (77)	9.8% (47)	6% (29)	7.5% (36)
	t2	15.3% (73)	17.4% (83)	17.6% (84)	18.9% (90)	10.3% (49)	8.4% (40)	5.9% (28)	6.3% (30)

**Table 5:** Spearman correlations of smartphone usage, physical activity, and mental health outcomes at t2.

	Girls		Boys	
Variable	1. Smartphone Use	2. Physical Activity	1. Smartphone Use	2. Physical Activity
1. Smartphone use				
2. Physical activity	−0.221 **		−0.144	
3. General anxiety	0.135 **	−0.192 **	0.160	−0.213 *
4. Depression	0.214 **	−0.164 **	0.335 **	−0.077
5. Insomnia	0.185 **	−0.171 **	0.353 **	−0.136
6. Well-being	−0.213 **	0.264 **	−0.235 *	0.235 **
7. Stress	0.144 **	−0.170 **	0.311 **	−0.292 **

* indicates *p* < 0.05, ** indicates *p* < 0.01.

## Data Availability

The raw data supporting the conclusion of this article will be made available by the authors upon reasonable request.

## Supplementary Materials

The following supporting information can be downloaded at: https://www.mdpi.com/article/10.3390/ijerph192013110/s1.
