# Supplementary material for: Assessment of the Long-Term Mental Health Effects on Austrian Students after COVID-19 Restrictions
Source: Int J Environ Res Public Health. 2022 Oct 12;19(20):13110. doi: 10.3390/ijerph192013110 (PMC9603217; doi:10.3390/ijerph192013110)

Supplementary Tables

Supplementary Table S1. Descriptive statistics of mental health outcomes for diverse adolescents.

Totall	Diverse	
	**t1**	**t2**
**N**	50	17
**WHO-5** *M (SD)*	20.96 (15.83)	20.47 (16.06)
**PHQ-9** *M (SD)*	18.24 (5.39)	18.53 (6.02)
≥11, *%* (N)	94 (47)	94.1 (16)
**GAD-7** *M (SD)*	14.22 (4.92)	13.53 (6.02)
≥11, *%* (*N*)	72 (36)	70.6 (12)
**ISI** *M (SD)*	12.38 (4.87)	14.00 (5.57)
≥15, *%*(*N*)	30 (15)	41.2 (7)
**PSS-10** *M (SD)*	29.44 (7.31)	26.35 (7.79)
≥14, *%* (*N*)	94 (47)	88.2 (15)

Note: t1: Sample from February 2021 (after one semester of remote schooling).t2:Sample from April-May 2022 (school opened and most restrictions lifted)

Table S2. General Linear Model for mental health outcomes with predictors gender, time and gender*time interaction

Criterion	Predictor	*b*	*SE*	*t*	*p*	*Adj.r²*
**WHO-5**	Intercept	36.62	0.89	41.251	<.001	.04
	Time (Study)	-3.94	1.26	-3.128	<.001	
	Gender	7.99	2.00	3.993	<.001	
	Time*gender	3.36	2.81	1.195	.233	
**PHQ-9**	Intercept	11.99	0.28	42.477	<.001	.06
	Time (Study)	2.17	0.40	5.419	<.001	
	Gender	-2.80	0.64	-4.408	<.001	
	Time*gender	-0.66	0.89	-0.735	.462	
**GAD-7**	Intercept	10.80	0.2316	46.648	<.001	.05
	Time (Study)	0.68	0.3282	2.077	.038	
	Gender	-2.75	0.5218	-5.274	<.001	
	Time*gender	-0.16	0.7333	-0.239	.811	
**ISI**	Intercept	10.85	0.2646	41.022	<.001	.03
	Time (Study)	1.03	0.3750	2.759	<.001	
	Gender	-2.59	0.5962	-4.347	<.001	
	Time*gender	0.25	0.8379	0.301	.763	
**PSS-10**	Intercept	24.35	0.3169	76.827	<.001	.06
	Time (Study)	0.55	0.4491	1.225	0.221	
	Gender	-4.14	0.7140	-5.804	<.001	
	Time*gender	-0.73	1.0035	-0.726	0.468	

Supplementary Table S3. Statistical results for comparisons between t1 and t2 for boys and girls.

	t1[a] vs t2[b]		t1 vs t2	
	Girls		Boys	
	**χ^2(df,N), McNemar's OR [95%CI]**	***p***	**χ^2(df,N), McNemar'sOR [95%CI]**	***p***
**PHQ-9**				
≥11	χ^2*(1,958)=*15.45,*OR=*1.78 [1.34,2.44]	<.001	χ^2*(*1,240)=0.955,*OR*=1.31[.79,2.26]	.328
**GAD-7**				
≥11	χ^2(1,958)=4.22,*OR*=1.34 [1.02,1.80]	.022	χ^2(1,240)=.017,OR=1 [.57,1.76]	.896
**ISI**				
≥15	χ^2(1,958)=10.05,OR=1.63 [1.21,2.25]	.002	χ^2(1,240)=.432,OR=1.31 [.65,2.86]	.511
**PSS-10**				
≥14	χ^2(1,958)=2.82,OR=1.61 [.99,2.82]	.093	χ^2(1,240)=.000,OR=1.06[.502,2.27]	.999
**Suicidal Ideation**				
PHQ9 Item9: ≥3	χ^2(1,958)=7.81,OR=1.96 [.1.24,3.39]	.005	na*	
**Excessive Smartphone use**				
≥5h/day	χ^2(1,958)=8.822,OR=0.68 [.527,.878]	<.001	χ^2(1,240)=13.99,OR=0.37[219,.627]	<.001

Note: [a] t1: Sample from February 2021 (after one semester of remote schooling).[b] Sample from April-May 2022 (school opened and most restrictions lifted). *t(df):* test-statistic of the t-test (degrees of freedom); d: Effect size Cohen's d; χ^2=test-statistic of the χ^2-Test; OR: Odds Ratio; CI: Confidence Interval: p-value (2-tailed, for χ^2 1-tailed). *na: calculation of Mc-Nemars OR not possible due to zero events in cells.

Supplementary Table S4. Spearman correlations of smartphone usage and mental health outcomes in girls at t1.

Spearmans Rank Correlation Coefficients (rho) with confidence intervals

Variable	1. Smartphone Usage	2. Physical Activity
1. Smartphone Usage		
2. Physical Activity	-.289**	
3. GAD7-score	.236**	-.169**
4. PHQ9-score	.271**	-.194**
5. ISI-score	.315**	-.190**
6. WHO5-score	-.279**	.297**
7. PSS10-score	.228**	-.250**

Note. t1: Sample from February 2021 (after one semester of remote schooling).

Values in square brackets indicate the 95% confidence interval for each correlation. The confidence interval is a plausible range of population correlations that could have caused the sample correlation (Cumming, 2014). * indicates $p < .05$. ** indicates $p < .01$.

Supplementary Table S5. Spearman correlations of smartphone usage and mental health outcomes in boys at t1.

Spearmans Rank Correlation Coefficients (rho) with confidence intervals

Variable	1. Smartphone Usage	2. Physical Activity
1. Smartphone Usage		
2. Physical Activity	-.325**	
3. GAD7-score	.416**	-.303**
4. PHQ9-score	.433**	-.367**
5. ISI-score	.294**	-.252
6. WHO5-score	-.361**	.424**
7. PSS10-score	.338**	-.326**

Note. t1: Sample from February 2021 (after one semester of remote schooling).
Values in square brackets indicate the 95% confidence interval for each correlation. The confidence interval is a plausible range of population correlations that could have caused the sample correlation (Cumming, 2014). * indicates $p < .05$. ** indicates $p < .01$.

Supplementary Table S6. Differences in correlation coefficients between t1 and t2 in girls.

Variable	1	2
1. Smartphone Usage		
2. Physical Activity	Z=-1.123 *p*=0.131	
3. GAD7-score	*Z=1.615* *p*=0.053	Z=0.367 *p*=0.357
4. PHQ9-score	Z=0.935 *p*=0.175	Z=-0.478 *p*=0.316
5. ISI-score	Z=2.143 *p*=0.016	Z=2.143 *p*=0.016
6. WHO5-score	Z=-1.084 *p*=0.139	Z=-1.387 *p*=0.083
7. PSS10-score	Z=1.343 *p*=0.09	Z=1.292 *p*=0.098

Note. t1: Sample from February 2021 (after one semester of remote schooling), t2:Sample from April-May 2022 (school opened and most restrictions lifted).

Supplementary Table S7. Differences in correlation coefficients between t1 and t2 in boys.

Variable	1	2
1. Smartphone usage		
2. Physical activity	Z=-1.47 *p*=0.071	
3. GAD7-score	*Z=2.152* *p*=0.016	Z=-0.738 *p*=0.23
4. PHQ9-score	Z=0.88 *p*=0.189	Z=-2.354 *p*=0.009
5. ISI-score	Z=-.504 *p*=0.307	Z=-0.923 *p*=0.178
6. WHO5-score	Z=-1.06 *p*=0.145	Z=1.63 *p*=0.052
7. PSS10-score	Z=0.231 *p*=0.409	Z=0.288 *p*=0.387

Note. t1: Sample from February 2021 (after one semester of remote schooling), t2:Sample from April-May 2022 (school opened and most restrictions lifted).

Supplementary Figure S1. Covariance balance of unmatched vs. matched samples

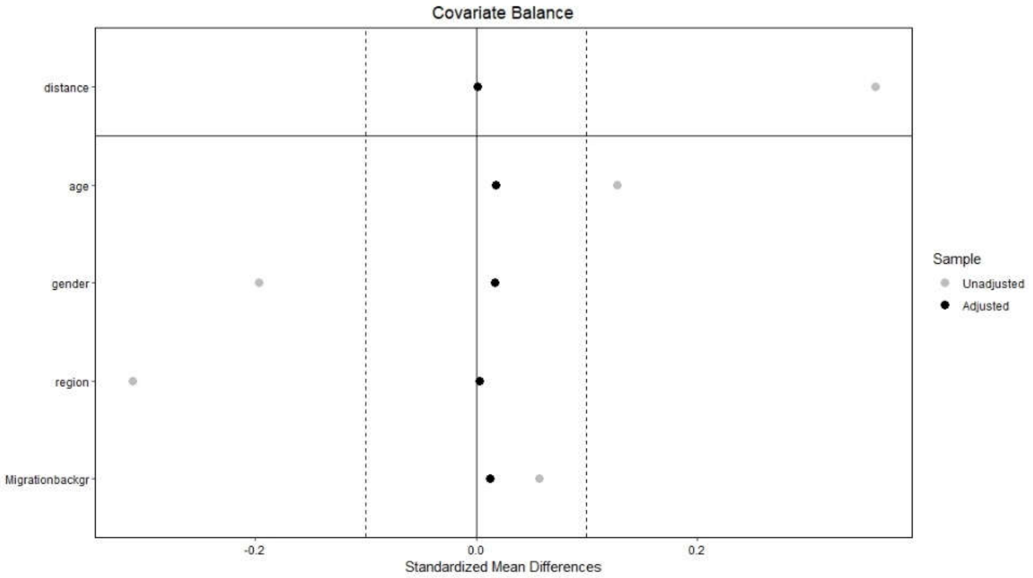

Supplement: Supplementary file 1 [file ijerph-19-13110-s001.zip › ijerph-1921820-supplementary.pdf]
